# Supplementary material for: Timeliness of routine childhood vaccination among 12–35 months old children in The Gambia: Analysis of national immunisation survey data, 2019–2020
Source: PLoS One. 2023 Jul 21;18(7):e0288741. doi: 10.1371/journal.pone.0288741 (PMC10361478; doi:10.1371/journal.pone.0288741)
Supplement: S1 Fig — (DOCX) [file pone.0288741.s001.docx]

# Supplementary material

**S1 Figure**: Flowchart displaying children included in this study by age group and birth/vaccination data completeness from the Gambia DHS 2019-2020 for computing timeliness.

**5,148** children aged **0-35 M** in DHS data had immunization data

**1586** children aged **24-35 M** included in **crude coverage** analysis

**12 -23 Months**

The **number and proportion** (out of 1662) of children with valid dates on their vaccination card who were ***included in the timeliness analysis***

**HepB0** = 1460 (87.8%)

**BCG** = 1459 (87.8%)

**OPV1** = 1456 (87.6%)

**OPV2** = 1441 (86.7%)

**OPV3** = 1417 (85.3%)

**PENTA1** = 1457 (87.7%)

**PENTA2** = 1442 (86.8%)

**PENTA3** = 1419 (85.4%)

**MCV1** = 1363 (82.0%)

**3,248** children were **12-35 M*** and included in the analysis

**1662** children aged **12-23 M** included in **crude coverage** analysis

**24 -35 Months**

The **number and proportion** (out of 1586) of children with valid dates on their vaccination card who were ***included in the timeliness analysis***

**HepB0** = 1265 (79.8%)

**BCG** = 1269 (80.0%)

**OPV1** = 1269 (80.0%)

**OPV2** = 1264 (79.7%)

**OPV3** = 1240 (78.3%)

**PENTA1** = 1274 (80.3%)

**PENTA2** = 1267 (79.9%)

**PENTA3** = 1244 (78.4%)

**MCV1** = 1218 (76.8%)

***Note:** This analysis was restricted to the 12-35 months age group to ensure that the timeliness estimates is comparable to the crude vaccination coverage rates which are published by the DHS survey.
