# Supplementary material for: Timeliness of routine childhood vaccination among 12–35 months old children in The Gambia: Analysis of national immunisation survey data, 2019–2020
Source: PLoS One. 2023 Jul 21;18(7):e0288741. doi: 10.1371/journal.pone.0288741 (PMC10361478; doi:10.1371/journal.pone.0288741)
Supplement: S1 Table — (DOCX) [file pone.0288741.s002.docx]

**S1 Table:** Median number of days children were vaccinated too early and interquartile ranges for all vaccines for children 12-23 and 24-35 months in The Gambia

|  | **12 - 23 Months** | | | **24 - 35 Months** | | |
| --- | --- | --- | --- | --- | --- | --- |
| **Vaccines** | **Median** | **1st quartile** | **3rd quartile** | **Median** | **1st quartile** | **3rd quartile** |
| **OPV1** | 7 | 2 | 17 | 8 | 2 | 18 |
| **OPV2** | 3 | 2 | 12 | 5 | 2 | 13.5 |
| **OPV3** | 4 | 2.3 | 12 | 5.5 | 3 | 13.5 |
| **PENTA1** | 6 | 2 | 15.8 | 8 | 2 | 17 |
| **PENTA2** | 3 | 1 | 11 | 4.5 | 2 | 13.8 |
| **PENTA3** | 4 | 2 | 10 | 5 | 3 | 13 |
| **MCV1** | 14.5 | 6.8 | 26.3 | 9 | 4 | 26 |

**Note:** Pentavalent vaccine (DPT-HepB-Hib); OPV = Oral Polio Vaccine; MCV = Measles Containing Vaccine
