# Supplementary material for: Timeliness of routine childhood vaccination among 12–35 months old children in The Gambia: Analysis of national immunisation survey data, 2019–2020
Source: PLoS One. 2023 Jul 21;18(7):e0288741. doi: 10.1371/journal.pone.0288741 (PMC10361478; doi:10.1371/journal.pone.0288741)
Supplement: S2 Table — (DOCX) [file pone.0288741.s003.docx]

**S2 Table**: Median delays and interquartile ranges for all vaccines for children 12-23 and 24-35 months in The Gambia

|  | **12 - 23 Months** | | | **24 - 35 Months** | | |
| --- | --- | --- | --- | --- | --- | --- |
| **Vaccine** | **Median** | **1st quartile** | **3rd quartile** | **Median** | **1st quartile** | **3rd quartile** |
| **HepB0** | 16 | 9 | 26 | 17 | 10 | 28 |
| **BCG** | 11 | 5 | 19.5 | 13 | 6 | 21 |
| **OPV1** | 14 | 5 | 26 | 11 | 4 | 29 |
| **OPV2** | 20 | 8 | 35.3 | 22 | 9 | 45 |
| **OPV3** | 26 | 11 | 52 | 28 | 11 | 57 |
| **PENTA1** | 15 | 5 | 26.5 | 12 | 4 | 30 |
| **PENTA2** | 21 | 8 | 37 | 22 | 9 | 45 |
| **PENTA3** | 26 | 11 | 51 | 27 | 11 | 55 |
| **MCV1** | 20 | 8 | 42 | 22 | 9 | 48.8 |

**Note:** HepB0 = Birth dose of Hepatitis B vaccine; BCG = Bacille Calmette-Guérin; PENTA = Pentavalent vaccine (DPT-HepB-Hib); OPV = Oral Polio Vaccine; MCV = Measles Containing Vaccine
